# Joan Bicknell

**DOI:** 10.1192/bjb.2017.21

**Published:** 2018-02

**Authors:** Sheila Hollins

Dorothy Joan Bicknell, psychiatrist, born 10 April 1939; died 12 June 2017

**Figure:**
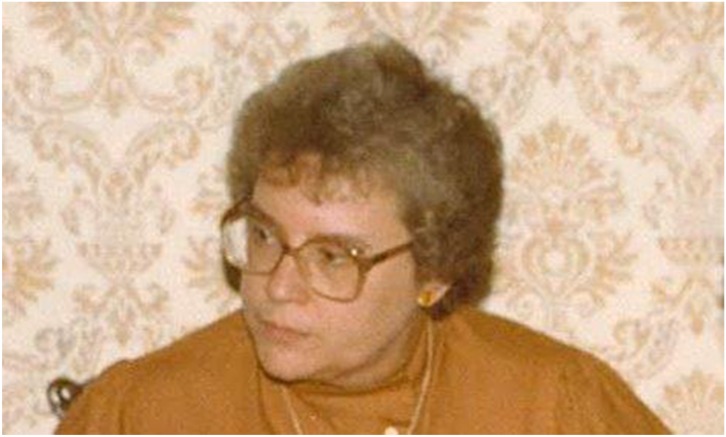
Brilliant clinician who was appointed professor of psychiatry of learning disability at St George's, University of London.

In 1980, Joan Bicknell, who has died aged 78 of cancer, was appointed professor of psychiatry of learning disability – the first British female professor of psychiatry – at what is now St George's, University of London. A brilliant clinician, she put the disabled person and their family at the centre of each consultation.

She was in constant demand to speak to trainees, parent groups and learning disability services about her vision of what could be done, and published dozens of papers for both clinicians and families. An article drawn from her inaugural lecture as a professor in 1981, The Psychopathology of Handicap, was published in the journal Psychological Medicine, and remains inspirational today.

In it, she described the emotional effect on parents and siblings of the diagnosis of learning disability in the family and also explored what it is like for children and adults living with a learning disability in terms of their own emotional inner world, their expectations for adulthood and the extent to which they were included or excluded from participating in daily life in the community. At the time, most people with learning disabilities lived at home with their families – unless the family could no longer support the person – and the alternative was usually a hospital admission.

Joan demonstrated that with specialist advice, community services could provide families with more support to care for longer, and the growing number of community homes and hostels could be supported to provide family-style care. This was unpopular with many medical superintendents of “mental handicap” hospitals, and she was never accepted by the medical establishment.

In 1978, a public inquiry into a scandal at Normansfield hospital in Teddington, south-west London, found the medical leadership seriously at fault. The nurses at Normansfield had gone on strike two years earlier complaining about how dysfunctional the organisation was, and the appalling conditions in which people with learning disabilities were living. The South West Thames regional health authority accepted the inquiry recommendations and appointed Joan to lead a taskforce to transform the care provided. It brought staff and relatives together to create an improvement plan, a new multidisciplinary management structure was put in place and money was provided to build more accommodation to reduce overcrowding.

It was against this background that Joan became professor, heading a new NHS-funded academic department to launch the specialty of psychiatry of learning disability at St George's, covering south-west London, Surrey, Sussex and Hampshire. St George's remains a leader in teaching medical students how to communicate effectively with people with learning disabilities, work that began in 1982, when Joan started involving her patients and their families in face-to-face teaching.

During her working day she met patients and families in the clinic or at home, supervised trainees, advised NHS and social service managers and fielded phone calls from people seeking advice. In the evenings and at weekends she would be booked as a speaker or for consultations all over Britain. She took early retirement in 1990 at the age of 50, having suffered from mental illness and severe asthma for the final three years of her career.

Perhaps the hardest thing for her to deal with had been the failure of NHS managers and of politicians to respond with meaningful action when she spoke up for the human rights of people with learning disabilities. She wrote in the Personal View column for the British Medical Journal about the men in a locked ward who were not allowed access to the kitchen or to drinking water and resorted to filling their shoes with water from the lavatory pan. Eventually a water fountain was installed in the living room, only for it to be blocked up by staff using it to dispose of their cigarette ash. Confronting cruelty in such hospitals was yet another of the ways she contributed to the advance of enlightened disability psychotherapy and psychiatry.

Born in Isleworth, south-west London, Joan lived in the capital for most of the second world war with her mother, Dorothy (nee Smith), a solicitor's secretary and later a foster parent, and her older brother, Edward, who went on to become a teacher in Swaziland, where he later died. Her father, Albert Bicknell, a foreman bricklayer in peacetime, served with the Royal Engineers in bomb disposal.

From Twickenham County school for girls Joan went to Birmingham University, graduating in medicine in 1962. A Methodist missionary society needed a paediatrician urgently at the Ilesha Wesley Guild hospital in Oyo, Nigeria, so Joan interrupted her paediatric training to test her new medical skills in a very different setting. When the Biafran war began in 1967, Joan was rounded up with others at bayonet point and put on a plane to Sierra Leone. There she worked on the flying doctor service before returning home.

She took a post at Queen Mary's hospital, Carshalton, in Surrey, where her concern about the poor care given to severely disabled children was strengthened by her own family experience, when her mother fostered two brothers with learning disabilities. This was the new challenge she needed and five more years of clinical training followed.

Joan obtained the diploma in psychological medicine in 1969, and in 1971 completed an MD on the causes and prevalence of lead poisoning in institutionalised children. She was soon active in campaigning about the restricted and poorly supported lives that people experienced in long-stay hospitals.

In 1972 Joan became a consultant psychiatrist in mental handicap at Botley's Park hospital, Chertsey, where she later met Diane Worsley, a social worker. Their friendship and partnership stood the test of time, and after Joan retired they moved to Holnest, Dorset, and ran a farm, welcoming disabled children to work with the animals. Joan and Diane also built up the Longburton Methodist chapel, where they were worship leaders. In 2016 they sold the farm and moved to nearby Stalbridge.

The community team base at Springfield University hospital in Tooting, south-west London, where Joan did much of her pioneering clinical work, was named after her when she left. The Royal College of Psychiatrists instituted an annual essay prize in her honour.

She is survived by Diane.

## Notes

This article has been reproduced with kind permission from theguardian.com.

